# The Nomogram of Penile Length and Circumference in Iranian Term and Preterm Neonates

**DOI:** 10.3389/fendo.2018.00126

**Published:** 2018-05-04

**Authors:** Fahimeh Soheilipour, Farzaneh Rohani, Elham Hashemi Dehkordi, Roya Isa Tafreshi, Parisa Mohagheghi, Seyed-Mohammadsalar Zaheriani, Fatemeh Jesmi, Hamid Salehiniya

**Affiliations:** ^1^Pediatric Growth and Development Research Center, Institute of Endocrinology and Metabolism, Iran University of Medical Science, Tehran, Iran; ^2^Child Growth and Development Research Center, Research Institute for Primordial Prevention of Non-Communicable Disease, Isfahan University of Medical Sciences, Isfahan, Iran; ^3^Department of Pediatrics, Iran University of Medical Sciences, Tehran, Iran; ^4^Minimally Invasive Surgery Research Center, Iran University of Medical Sciences, Tehran, Iran; ^5^Pars Advanced and Minimally Invasive Medical Manners Research Center, Pars Hospital, Iran University of Medical Sciences, Tehran, Iran; ^6^Zabol University of Medical Sciences, Zabol, Iran; ^7^Department of Epidemiology and Biostatistics, School of Public Health, Tehran University of Medical Sciences, Tehran, Iran

**Keywords:** nomogram, Iran, penis agenesis, regression analysis, stretched penile length, penile circumference

## Abstract

**Background and objectives:**

The normal length of penis in preterm and term neonates is different among different nations, and is affected by various factors. The present study aimed to determine stretched penile length (SPL) values and cutoff level of micropenis in term and preterm Iranian neonates, for the first time.

**Materials and methods:**

All male neonates born in two general hospitals of Tehran (Akbarabadi, and Rasoul hospitals), center of Iran, with gestational age of 28–42 weeks were included and their SPL and penile circumference (PC) were examined on the first 3 days after birth by the same physician. Birth weight (BW), and height, gestational age, type of delivery, mother’s age, twin/multiple pregnancy, drug, and medical history of mother during pregnancy were recorded and cutoff levels of two variables were calculated based on the collected variables for different gestational ages.

**Results:**

Among a total of 587 neonates, 203 neonates were born term and 384 preterm. Mean ± SD of neonates’ BW were 2,682.51 ± 739.30 (850–4800) gr. Mean ± SD of their SPL was 22.48 ± 3.34 mm; 25.92 ± 1.54 mm in term and 20.66 ± 2.50 mm in preterm infants (*P* = 0.001). Mean ± SD of PC was 6.71 ± 1.31 mm; 8.14 ± 0.48 in term and 5.96 ± 0.92 in preterm infants (*P* = 0.001). SPL and PC were significantly correlated with type of delivery, number of parity, gestational age, BW, and crown-heel length, head circumference (*P* < 0.001).

**Conclusion:**

This study suggested that SPL in male neonates was 22.48 mm and PC was 6.71 mm, both correlated with gestational age and BW. Due to the ethnical variety of this cutoff points and lack of an appropriate study in Iran, these cutoff points can be used by all physicians as a reference for Iranian newborns (term and preterm), in order to prevent misdiagnosis of micropenis and genital disorders.

## Introduction

One of the important concerns in neonates’ physical examination is the genital examination, especially penile size that is considered an important representation of hypothalamic or pituitary abnormality (hypogonadotropic hypogonadism); due to the role of androgen exposure in fetal sex development, disorders in genital system may be a sign of disorders of sex development (DSDs) (1). Thus, penile measurement contributes to the diagnosis of the underlying genetic or endocrine disorders (2). In addition to diagnosis of hypothalamic and pituitary defects, measurement of PL is important in procedures, such as circumcision, as well (3).

Micropenis is defined as a small penis, without epispadias or hypospadias, 2.5 SDs below the mean PL (4). Pure micropenis, not associated with DSDs, benefit from early intervention, especially in minipuberty stage of below 6 months of age, when low dose testosterone can improve the penile length significantly (1, 5). Therefore, the accuracy of PL measurement and cutoff for the definition of micropenis are of great importance, commonly missed in early physical examination of the newborn (6). Stretched penile length (SPL) is basically put on nomograms, designed based on gestational age, weight, and height (7); accordingly, PL values are different for preterm infants (8), as well as different ethnicities (9). Therefore, it is necessary to measure neonate’s PL, according to the nomograms defined for that nationality, although currently many neonatologists, including Iranian specialists, use the international nomograms, which might misdiagnose micropenis.

As far as the authors are concerned, very few studies in Iran have investigated PL in Iranian infants. One study in Golestan province on 427 infants determined mean PLs in Fars, Turkmen, and Sistani infants, although they have not differentiated term and preterm infants and did not report SDs to set the micropenis cutoff (10). Considering various penoscrotal abnormalities, which remain undiagnosed until school age in Iran (11), determining the penile nomogram in Iranian infants is of great importance. Therefore, the present study aimed to determine mean and SD values of PL and penile circumference (PC) in term and preterm Iranian neonates and its correlation with different variables.

## Materials and Methods

### Study Design

In the present study, all male neonates born in two hospitals of Tehran (Akbarabadi, and Rasoul hospitals), Iran were included from March 2012 until June 2014 and their SPL were examined on the first 3 days after birth by two trained examiners.

Inclusion criteria consisted of live male neonates under 3 days (72 h) of age, with gestational age of 28–42 weeks, without congenital malformations and apparent genital anomalies, such as hypospadias, epispadias, and undescendent testis. Neonates who had greater than 2 weeks discrepancy in calculation of gestational age by first trimester ultrasound or last menstrual period by Ballard scoring system were excluded from the study. Neonates who met the inclusion/exclusion criteria were included into the study by convenient sampling method until saturation of sample size.

Data collected included infant’s ethnicity, SPL, birth weight (BW), and crown-heel length (CHL), head circumference (HC), gestational age considered by last menstrual period (GALMP), type of delivery, mother’s age, singleton/twin/multiple pregnancies, drug, and medical history [gestational diabetes and (pre)eclampsia] of mother during pregnancy.

Stretched penile length was measured in the supine position with flexed legs. For measurement of penis, the fat pad of symphysis pubis was pushed and measured from symphysis pubis to the tip of phallus glans in complete stretching by a digital caliper (Aesculap, Center Valley, PA, USA) (12). Examination was performed in 37°C room temperature and sufficient light. For measurement of neonate’s CHL, first the neonate was placed supine, without clothes, on an infantometer (Harpenden, London, UK), with 1-cm precision, and the head was held at the sign and knees were stretched to straighten the legs (13) and neonate’s weight was measured by a digital scale with a precision of 10 g. Gestational age was calculated by first trimester ultrasound or last menstrual period by Ballard scoring system.

Neonates born 2.5–4 kg were considered appropriate for gestational age, <2.5 kg as low BW which contain two categories: preterm neonate or small for gestational age, and >4 kg as large for gestational age.

### Ethical Considerations

The protocol of the study was approved by Ethics committee of Iran University of Medical Sciences. The design and objectives of the study were explained to the parents of all participants and written informed consent was obtained from those who were willing to participate their neonates into the study and were clarified that their data would be kept confidential and analyzed anonymously.

### Statistical Analysis

Results were presented as mean ± SD for quantitative variables and were summarized by frequency (percentage) for categorical variables. The correlation of variables was tested by correlation analysis and backward regression analysis was used for prediction penile length and circumference. For the statistical analysis, the statistical software SPSS version 16.0 for windows (SPSS Inc., Chicago, IL, USA) was used. *P* values of 0.05 or less were considered statistically significant.

## Results

Among a total of 587 neonates, 203 neonates were born term (≥38 weeks) and 384 were born preterm (<38 weeks). Mean mother’s age of the included neonates were 25.59 ± 4.88 (range: 14–40) years. Among all participants, 37.5% (*N* = 220) were born by cesarean section and 62.5% (*N* = 367) by vaginal delivery.

Mean ± SD of neonates’ BW were 2,682.51 ± 739.30 (850–4,800) gr. The frequency of gestational diabetes, and/or (pre)eclampsia (during mother’s pregnancy) was 10.7%. A total of 85.2% were singleton pregnancies, 11.1% were twin pregnancies, and 3.7% were triplets.

Mean ± SD of SPL were 25.92 ± 1.54 mm in term infants and 20.66 ± 2.50 mm in preterm infants (*P* = 0.001). Mean ± SD of PC was 8.14 ± 0.48 in term infants and was 5.96 ± 0.92 in preterm infants (*P* = 0.001).

**Figure 1:**
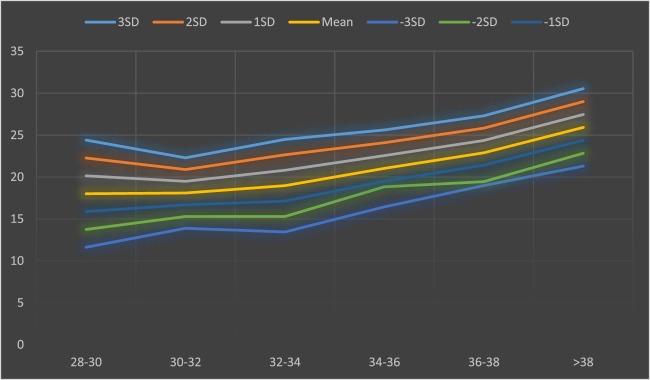
Mean and SD of penile length based on different gestational age of neonates.

**Figure 2:**
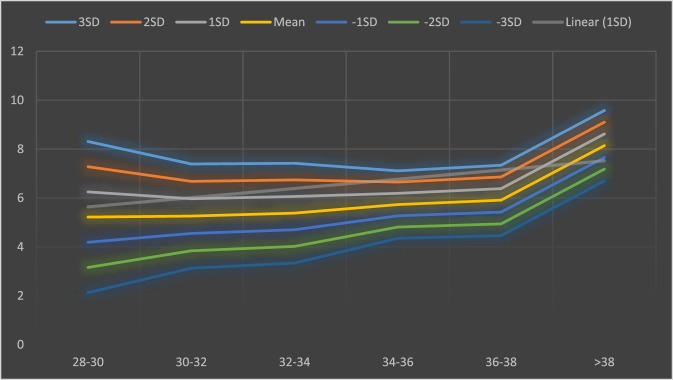
Mean and SD of penile circumference (PC) based on different gestational age of neonates.

The lower and upper limits (±3 SD) of SPL for total, term, and preterm infants were 32.5–12.46, 30.54–21.3, and 28.16–13.16 mm, respectively, and that of PC were 10.46–2.78, 9.58–6.7, and 8.72–3.2 mm, respectively. Details of ±1SD, ±2SD, and ±3SD of PC and length are demonstrated in Tables 1 and 2, based on different gestational age of neonates (Figures 1 and 2).

**Table 1:** Mean and SD of penile length based on different gestational age of neonates.

Gestational age, week	Number	3SD	2SD	1SD	Mean	−3SD	−2SD	−1SD
28–30	31	24.40	22.27	20.14	18.01	11.62	13.75	15.88
30–32	50	22.29	20.89	19.49	18.09	13.89	15.29	16.69
32–34	71	24.49	22.65	20.81	18.97	13.45	15.29	17.13
34–36	102	25.62	24.09	22.56	21.03	16.44	18.83	19.50
36–38	130	27.3	25.83	24.36	22.89	18.98	19.45	21.42
>38	203	30.54	29	27.46	25.92	21.3	22.84	24.38

**Table 2:** SD of penile circumference (PC) based on different gestational age of the study neonates.

Gestational age, week	Number	3SD	2SD	1SD	Mean	−1SD	−2SD	−3SD
28−30	31	8.31	7.28	6.25	5.22	4.19	3.16	2.13
30−32	50	7.39	6.68	5.97	5.26	4.55	3.84	3.13
32−34	71	7.42	6.74	6.06	5.38	4.7	4.02	3.34
34−36	102	7.11	6.65	6.19	5.73	5.27	4.81	4.35
36−38	130	7.34	6.86	6.38	5.91	5.42	4.94	4.46
>38	203	9.58	9.1	8.62	8.14	7.66	7.18	6.7

Correlation analysis showed that penile length was correlated with type of delivery (*r* = 0.349), number of neonates in each pregnancy (*r* = −0.42), GALMP (*r* = 0.85), BW (*r* = 0.77), CHL (*r* = 0.73), and HC (*r* = 0.73) and not with other variables, such as mother’s age and parity (Table 3), and PC was correlated associated with type of delivery (*r* = 0.32), number of neonates in each pregnancy (*r* = −0.42), GALMP (*r* = 0.84), BW (*r* = 0.77), CHL (*r* = 74), and HC (*r* = 0.71) and not with other variables, such as mother’s age and parity (Table 3).

**Table 3:** Correlation of penile length and penile circumference (PC) with other variables.

		Type of delivery	Number of neonates in each pregnancy	Mothers age	Gestational age considered by last menstrual period	Birth weight	HL	Head circumference
Penile length	Pearson correlation (*r*)	0.349**	−0.420**	0.056	0.848**	0.771**	0.723**	0.729**
	*P*-value	0.000	0.000	0.177	0.000	0.000	0.000	0.000
PC	Pearson correlation (*r*)	0.323**	−0.422**	−0.007	0.836**	0.773**	0.737**	0.706**
	*P*-value	0.000	0.000	0.865	0.000	0.000	0.000	0.000

**Correlation is significant at 0.01 (two-tailed).

Correlation analysis showed that SPL was correlated with circumference (*r* = 0.884, *p* < 001). Backward regression analysis showed that SPL was associated with mother’s age, type of delivery, BW, HL, and HC (Penile length = 8.089 + 0.38 type of delivery + 0.28 mother’s age + 0.061 HL + 0.206 HC + 0.419 BW), also PC was associated with BW, HL, and HC (PC = 1.56 + 0.048 HL + 0.051 HC + 0.137 BW).

## Discussion

The present study determined mean and SD values of SPL and PC in term and preterm Iranian neonates and its correlation with different variables. As the results indicated, mean ± SD of SPL and PL were 22.48 ± 3.34 and 6.71 ± 1.31 mm, respectively, in the total population of term and preterm infants. Then, ±1, ±2, and ±3 SD were defined for each group.

In a similar study in Iran, Golestan province, mean ± SD of PL was reported 32.1 ± 3.5 mm (10), which is higher than SPL of term infants in the present study (25.92 ± 1.54 mm), although difference in the technique of measurement and possible observer bias can play a role in such a difference, in addition to the fact that the infants of the present study did not vary in ethnicity, while Alaee et al. had selected infants of three different ethnical groups, including Fars, Turkmen, and Sistani. Nevertheless, the mean value of SPL in the present study is not lower than the cutoff they have set for micropenis (<23.3 mm), which shows the consistency of the results of the different studies, but the cutoff they have set is higher than the −2 and −3 SD in the present study (22.8 and 21.3, respectively).

As we know, the reference values that pediatricians usually use are related to many years ago and based on the results of small sample sizes (14), more importantly they are not specifically measured for Iranian population. Pediatricians and endocrinologist of the present study also considered the widely accepted cutoff of 20 mm for examination of term infants, as we did not have the results of this study at the time of measurement. As far as we are concerned, preterm infants are rarely investigated appropriately and, in the present study, we determined values for preterm infants as well. In measurements in 203 full-term and 384 preterm infants in the present study by this cutoff level, none of the neonates had abnormal values and, thus, none required further examination, although we routinely refer neonates suspected of micropenis to the endocrinologist, who conventionally prescribes low dose testosterone for them and according to our >10 years’ experience, neonates appropriately respond to this treatment and their penile length improves significantly, although in our total samples, none had such problem.

Studies from different Asian countries report various results; a Turkish study (15), as well as a Chinese study (16), reported the mean SPL of term newborns at 30 mm with a cutoff of 22 mm for micropenis, which are higher than the results of the present study, while several reports from other countries state a mean SPL of >35 mm* (17), which seem much higher than that of the present study; an Indonesian studies report a mean SPL of 23.5 mm (18), which is lower than that of the present study, which can be attributed to the ethnical/racial differences. Another study reported the cutoff for micropenis (−2.5 SD) at 26, 25, and 23 mm for Caucasian, East-Indian, and Chinese newborns (19); thus, as SPL and the cutoff values are significantly different among various race/ethnicities, it is essential that neonatologists health staff of each country refer to the values relevant for their country, specifically for their special race.

In the present study, the relevant values for Fars term and preterm neonates with the normal variations have been determined. Moreover, most centers have measured both SPL and PC simultaneously (20); instead, a number of studies have investigated anogenital distance (AGD) that had a significant correlation with SPL, because it is another important index in the initial measurements of penis (10, 21). A recent study in Ghana has reported penile width as an important variable while studying penile size, which is in favor of our results, although the mean value they have reported (10 mm) (20) seems higher than mean PC in term neonates of the present study (8 mm), which is much closer to the value reported from Indonesia (22). Yet, as ethnicity play an important role in variation of PC, comparison of studies from different countries is not possible, and further Iranian studies are required in this regard, as the present study, to the best of our knowledge, was the first Iranian study to measure PC in a large population of neonates.

Another important finding of the current study is the correlation of SPL and PC with number of GALMP, BW, CHL, and HC of neonates. Different researchers from many countries also confirmed the association of SPL with anthropometric variables (9, 15). The linear association of BW with SPL, as confirmed in numerous studies (23, 24), can be justified by the simultaneous growth mechanisms of fetal body size (BW, HC, HL) and penile length and circumference through hormonal balance (25). This suggests further attention of gynecologists to maintain appropriate BW of infants during pregnancy for prevention of micro/macropenis. According to the formula suggested, SPL could be predicted with specific coefficients by type of delivery, mother’s age, HC, and BW.

Other researchers have justified that this association is also affected by ethnicity of the newborn that may cause different results (26). Yet, most studies are in line with the present study in this regard. The association of SPL with anthropometric variables and BW of newborns highlights this fact that SPL can be very variable in each population; thus, it is essential to refer to the normal value that defined for specific population and the results of the present study can help Iranian physicians and researchers in this respect.

Although SPL has been measured in multiple studies worldwide, the normal variations have been scarcely studied, especially in Iran, which is the main strength of the present study. However, the present study had also some limitations. As SPL is associated with various factors that might have not been covered in the present study, there is a possible effect of confounding factors on the results of the study, such as AGD, and time of circumcision, although we tried to investigate as many relevant variable as possible, such as BW, GA, mother’s age, and pregnancy-related diseases.

In conclusion, the results of the present study on 587 neonates (term and preterm) suggested the first smoothed percentile values (±1, 2, and 3SD) for SPL and circumference by gestational age that can be used as a reference for Iranian newborns.

## Ethics Statement

The protocol of the study was approved by Ethics committee of Iran University of Medical Sciences. The design and objectives of the study were explained to the parents of all participants and written informed consent was obtained from those who were willing to participate their neonates into the study and were clarified that their data would be kept confidential and analyzed anonymously.

## Author Contributions

FS: Data collection and interpretation, writing the manuscript, critical reviews, and response to reviewers. FR: Data collection, reviewing the article. ED: Data collection, reviewing the article. RT: Data collection, reviewing the article. S-MZ: Data collection. FJ: Data interpretation, writing the manuscript, critical reviews, and response to reviewers. HS: Data analysis, interpretation, writing the manuscript, critical reviews, and response to reviewers. PM: Data collection, reviewing the article. All authors: Proof of the latest version.

## Conflict of Interest Statement

The authors declare that the research was conducted in the absence of any commercial or financial relationships that could be construed as a potential conflict of interest.

## Abbreviations

BW, birth weight; CHL, crown-heel length; GALMP, gestational age considered by last menstrual period; HC, head circumference; LGA, as large for gestational age; PC, penile circumference; SGA, small for gestational age; SPL, stretched penile length; SD, standard deviation.
